# Editorial Comment: Renal Stone Features Are More Important Than Renal Anatomy to Predict Shock Wave Lithotripsy Outcomes: Results from a Prospective Study with CT Follow-Up

**DOI:** 10.1590/S1677-5538.IBJU.2020.02.04

**Published:** 2020-01-10

**Authors:** Alexandre Danilovic

**Affiliations:** 1 Hospital das Clínicas Faculdade de Medicina USP São PauloSP Brasil Serviço de Urologia, Hospital das Clínicas da Faculdade de Medicina da USP - HCFMUSP, São Paulo, SP, Brasil

Torricelli FCM ^1^, Monga M ^2^, Yamauchi FI ^1^, Marchini GS ^1^, Danilovic A ^1^, Vicentini FC ^1^, Batagello CA ^1^, Srougi M ^1^, Nahas WC ^1^, Mazzucchi E ^1^

^1^ Division of Urology, Hospital das Clinicas, University of Sao Paulo Medical School, Sao Paulo, Brazil; ^2^ Stevan B. Streem Center for Endourology & Stone Disease, Glickman Urological & Kidney Institute, The Cleveland Clinic, Cleveland, Ohio

J Endourol. 2020 Jan;34(1):63-67

**DOI:**10.1089/end.2019.0545 *|* **ACCESS:** 10.1089/end.2019.0545

## COMMENT

Predictors of shock wave lithotripsy (SWL) outcomes have been widely studied. Both renal stone features and collecting system anatomy are considered to play a major role to predict stone free rate of SWL. To date, EAU guideline recommends avoid SWL in lower pole kidney stone with an infundibulopelvic angle < 70°, an infundibular length > 30°, and an infundibular width ≤ 5 mm (1). However, those anatomical features were evaluated using intravenous pyelography, rarely used nowadays.

In this study, the authors used computerized tomography (CT) to challenge the concept of the importance of the lower pole location to the outcomes of SWL. In this prospective clinical study, the authors demonstrated the major predictors of SWL success were stone size, stone density and stone-skin distance but not stone location.

Interestingly, collecting system anatomy evaluated by CT seems to play a major role in flexible ureteroscopy (2) but not in SWL. In order to promote stone fragmentation, Holmium laser success depends more on collecting system anatomy but SWL depends more on stone features.

## References

[1] 1. Türk C, Petrík A, Sarica K, Seitz C, Skolarikos A, Straub M, et al. EAU Guidelines on Interventional Treatment for Urolithiasis. Eur Urol. 2016;69:475-82. 10.1016/j.eururo.2015.07.041 26344917

[2] 2. Danilovic A, Rocha BA, Torricelli FCM, Marchini GS, Batagello C, Vicentini FC, et al. Size is Not Everything That Matters: Preoperative CT Predictors of Stone Free After RIRS. Urology. 2019;132:63-68. 10.1016/j.urology.2019.07.006 31310774

